# Positional Convergence Spasm: A Case Report

**DOI:** 10.7759/cureus.82251

**Published:** 2025-04-14

**Authors:** Giorgi Mamardashvili, Nazibrola Botchorishvili, Sopiko Kartsivadze, Marina Janelidze

**Affiliations:** 1 Neurology, Tbilisi State Medical University, Tbilisi, GEO; 2 Neurology, S. Khechinashvili University Hospital, Tbilisi, GEO

**Keywords:** benign paroxysmal positional vertigo (bppv), positional convergence spasm, psychogenic convergence spasm, spasm of the near reflex, vertigo diagnosis

## Abstract

Benign paroxysmal positional vertigo is one of the most common causes of vertigo. Although quite bothersome, this condition can be easily treated using simple maneuvers. Sudden-onset diplopia, on the other hand, may indicate serious medical conditions. On rare occasions, diplopia may be related to psychogenic convergence spasm and can be induced by positional maneuvers.

We report the case of a patient presenting positional vertigo and brief episodes of diplopia without nystagmus, in whom psychogenic convergence spasm was provoked during positional testing. This rare phenomenon highlights the importance of considering functional etiologies in the differential diagnosis of atypical vertigo presentations to avoid unnecessary investigations.

## Introduction

Vertigo is a common symptom that can be associated with many different diseases. It can often be triggered by positional changes. One of the most frequent causes of positional vertigo is benign paroxysmal positional vertigo (BPPV), accounting for approximately 17-42% of vertigo cases [1]. Vertigo triggered by positional changes does not always mean BPPV [2]. Other diseases that cause positional vertigo may mimic BPPV [2]. The presence or absence of other accompanying symptoms can be key to differentiating it from other diseases.

Positional changes can provoke not only vertigo but also other symptoms such as transient diplopia [2]. The most common cause of sudden-onset diplopia is ischemic stroke, especially posterior circulation strokes such as basilar artery thrombosis, and must be carefully ruled out [3]. Apart from that, sudden-onset diplopia may be indicative of other neurological conditions such as myasthenia gravis, multiple sclerosis, ocular myopathies, third, fourth, and sixth nerve palsies [2]. Continuous or progressive diplopia with brainstem signs should make us think of central causes [3]. In contrast, intermittent symptoms without other neurological deficits may indicate a functional origin [4].

A less common but important differential is convergence spasm, which may also present with positional vertigo, mimicking BPPV [5]. The presence of vertigo with diplopia should always raise concern for potential serious underlying pathology [3]. Physicians encountering this combination should always consider stroke as the most frequent cause [3]. Once excluded, functional disorders such as convergence spasm should be included in the differentials [4].

In this case report, we describe a patient who presented with positional vertigo and brief episodes of diplopia. The clinical features and positional testing findings led us to diagnose psychogenic convergence spasm, highlighting the importance of recognizing functional disorders in patients with atypical vertigo symptoms.

## Case presentation

A 58-year-old female presented to a clinic with positional vertigo, instability, and episodes of transient diplopia. The first episode of positional vertigo occurred five days before admission upon getting up from bed. The following day, these symptoms resolved; however, a few hours before admission, with a background of emotional stress, she developed an episode of diplopia and could not move independently due to instability. The patient had no history of recent trauma or infection. Her medical history was significant for arterial hypertension, type 2 diabetes mellitus, and migraine without aura.

Neurological examination revealed that the patient was alert and fully oriented to the person, place, and time with normal speech. The pupils were reactive and symmetric, and visual fields were intact. Visual acuity was 20/20 bilaterally, and visual fields were full to confrontation. Oculomotor evaluation showed normal smooth pursuit and saccadic movements in all directions. There was no gaze palsy, nystagmus, or skew deviation. The cover-uncover test was normal. The vestibulo-ocular reflex (VOR) was intact, and the head impulse test was negative bilaterally. The test of skew showed no abnormalities.

No motor deficits were noted, and muscle strength was symmetrical bilaterally and graded 5/5. Sensation was preserved and symmetric on both sides. Magnetic resonance imaging (MRI) of the brain revealed essentially normal findings with no intra- or extra-axial space-occupying lesions. Laboratory tests and extracranial Doppler sonography revealed no abnormalities.

The patient was assessed using Frenzel goggles, which revealed no spontaneous or gaze-evoked nystagmus. However, during the Dix-Hallpike maneuver, the patient developed spontaneous convergence strabismus and the restricted ability to abduct the left eye with pupillary miosis and absence of nystagmus. This episode lasted 30-60 seconds and was accompanied by vertigo, diplopia and hyperventilation (Video 1).

**Video 1:** Positional Convergence Spasm Induced by Dix-Hallpike Maneuver with Left Eye Abduction Restriction, Miosis, No Nystagmus, and Associated Hyperventilation (30–60 Seconds) Signed consent was given by the patient for open-access publication of this video.

These findings were reproducible on repeated testing. Based on the patient’s presentation, treatment with trazodone and alprazolam was initiated, which led to the complete resolution of symptoms.

## Discussion

Convergence spasm, also called the spasm of the near reflex, is a clinical condition characterized by intermittent episodes of convergence, accommodation, and pupillary miosis. Normally, this reflex facilitates fixation on nearby objects [6]. However, when it persists even when the patient is not fixating on a near object, it is considered a spasm of the near reflex [6].

The differential diagnosis of convergence spasm is broad, it includes both organic and functional (psychogenic) causes [4,7]. Organic causes involve structural or metabolic disorders affecting the supranuclear pathways responsible for coordinating vergence and accommodation [8]. These include multiple sclerosis, Wernicke’s encephalopathy, encephalitis, or metabolic disorders [7]. Lesions affecting the dorsal midbrain, thalamomesencephalic junction, or occipital cortex have been implicated in cases of organic convergence spasm [8,9]. Mastroianni and Neri described a convergence spasm in a patient with focal occipital lobe epilepsy [10]. Emphasizing the diversity of possible underlying causes.

It can also mimic the abducens nerve palsy; convergence spasm presents with miosis, which is key to differentiating it from abducens nerve palsy [4].

It is important that convergence spasm may also have a psychogenic origin. Papageorgiou and Karadras reported 21 case of psychogenic convergence spasm, with over half of the patients exhibiting emotional or psychological stressors [11]. This aligns with our patient’s presentation. The episode was precipitated by emotional stress and resolved completely with Trazodone and Alprazolam treatment. The presence of reproducible, transient convergence spasm episodes in the absence of structural brain abnormalities on MRI or any other abnormal lab or imaging findings further supports a psychogenic etiology in this case [4,7].

Convergence spasm can mimic BPPV, particularly when episodes are triggered by positional changes [12]. Gordon and Almong described a patient exhibiting both BPPV and convergence spasms, emphasizing the fact that these conditions may present together [12]. In our case, the patient developed transient diplopia, vertigo, convergence strabismus, and left eye abduction restriction during the Dix-Hallpike maneuver. Notably, there was no associated nystagmus during the episode. The presence of pupillary miosis, absence of nystagmus, and reproduction of convergence spasm during positional testing indicate a non-vestibular cause of the symptoms [6].

Our patient’s condition turned out to be benign. However, the initial clinical picture of acute vertigo combined with diplopia should always make us think of potentially life-threatening central nervous system pathologies, such as brainstem or cerebellar stroke [3]. Acute vestibular syndrome, presenting with diplopia, dysarthria, or limb ataxia, requires emergent imaging to rule out vertebrobasilar ischemia [3].

In this case, evaluation of the patient with MRI of the brain, laboratory studies, and extracranial Doppler sonography ruled out structural or vascular pathology. Additionally, the absence of gaze-evoked or spontaneous nystagmus, direction-changing nystagmus, abnormal head impulse test, or other focal neurologic deficits made a central lesion less likely.

Based on the patient’s presentation, psychogenic convergence spasm was diagnosed, and the patient was administered with trazodone and alprazolam, which led to the complete resolution of symptoms.

## Conclusions

The first thing when encountering positional vertigo with transient diplopia is to rule out serious central nervous system pathologies such as brainstem or cerebellar strokes. In our case, normal imaging findings, with normal neurological examination, and absence of nystagmus led us to a non-organic etiology. The fact that symptoms were reproduced during positional testing, coupled with the resolution of symptoms after trazodone and alprazolam administration, supported the diagnosis of psychogenic convergence spasms. This case emphasizes the importance of considering functional disorders in the differential diagnosis of atypical presentations, but only after life-threatening conditions have been excluded.
